# Comparison in Sedative Effects between Dexmedetomidine and Midazolam in Dental Implantation: A Randomized Clinical Trial

**DOI:** 10.1155/2020/6130162

**Published:** 2020-06-02

**Authors:** Li Wang, Yi Zhou, Tiejun Zhang, Lili Huang, Wei Peng

**Affiliations:** ^1^The State Key Laboratory Breeding Base of Basic Science of Stomatology (Hubei-MOST) and Key Laboratory of Oral Biomedicine Ministry of Education, School and Hospital of Stomatology, Wuhan University, Wuhan, Hubei 430079, China; ^2^Department of Anesthesiology, School and Hospital of Stomatology, Wuhan University, Wuhan, Hubei 430079, China; ^3^Department of Prosthodontics, School and Hospital of Stomatology, Wuhan University, Wuhan, Hubei 430079, China

## Abstract

Dexmedetomidine refers to an *α*_2_-adrenergic receptor agonist causing potent sedative, analgesic, and minimal respiratory depression compared with alternative drugs. The present study was aimed at comparing the efficaciousness and safety of midazolam and dexmedetomidine as sedatives for dental implantation. We recruited 60 patients belonging to group I or II of the American Society of Anesthesiologists (ASA) and treated them with either midazolam or dexmedetomidine in a random manner. Patients' duration of analgesia after surgery, surgeon and patient degrees of satisfaction, Observer's Assessment of Alertness/Sedation Scale (OAAS) scores after drug administration, visual analogue scale (VAS) pain scores, and vital signs were recorded variables. Patients administered dexmedetomidine had significantly lower OAAS scores than those administered midazolam (*p* < 0.05). Patients administrated dexmedetomidine had a significantly longer analgesia duration after the surgical procedure than those administered midazolam, and the difference was statistically significant (*p* < 0.05). Dexmedetomidine had a significantly larger number of surgeons satisfied with the level of sedation/analgesia than midazolam (*p* < 0.05). Accordingly, it is considered that dexmedetomidine can achieve better postoperative analgesia, surgeon satisfaction, and sedation than midazolam.

## 1. Introduction

Dental implant is considered as the most common and popular treatment option for edentulous patients in modern dentistry. However, dental implants still have a significant relation to pain and high anxiety [1]. The role of sedation in dental surgery should not be overly highlighted. As pain and anxiety decrease, patients' cooperation and satisfaction increase [2]. Although various drugs have been used, “ideal” drugs and regimens have yet to be determined. Midazolam, a derivative of benzodiazepines, is widely used in dental sedation and is considered effective in reducing anxiety without causing cardiopulmonary instability [3]. It has been given in an oral or intravenous manner, either alone or together with propofol or opioids [4]. The sedation administered by a dentist or anesthesiologist was compared with the sedation administered by the patient [5]. Although it has been shown to be effective and reliable in sedation and has ideal anterograde amnesia properties, good surgical conditions, and satisfactory patients, it has close relation to delayed adverse respiratory effects, impairment of memory, and recovery and psychomotor function [6]. Dexmedetomidine refers to a highly selective agonist of an *α*_2_-adrenergic receptor. Its actions are considered mediated through postsynaptic *α*_2_ adrenoceptors which give activation to G proteins sensitive to the pertussis toxin. Activation of these receptors in the central nervous system results in effective sedation, analgesia, anxiety, and sympathetic effects, with minimal impact on respiratory physiological inhibition of sympathetic nerves [7]. Injecting small doses of the drug into healthy volunteers provides a sedative effect that can be easily reversed by verbal stimulation [8]. Dexmedetomidine is likely to be proven as a better sedative drug for dental sedation than midazolam for its lesser respiratory depression, lesser cognitive impairment, and shorter recovery profile and analgesic property. However, to our knowledge, dexmedetomidine has been rarely compared with midazolam for outpatient removal of dental implantation [9]. Our study is aimed at investigating dexmedetomidine's and midazolam's safety and efficacy (patients' satisfaction, operating conditions, analgesia, anxiolysis, and sedation) to be sedatives for dental implant surgery.

## 2. Patients and Methods

### 2.1. General Information

The Ethics Committee of the Hospital of Stomatology, Wuhan University, reviewed and approved this study prospectively ([2018] Ethical Review No. A [100]). Informed consent was obtained from the patients or their relatives. Patients who underwent dental implantation between January 2019 and December 2019 were enrolled. The study has been registered in the Chinese Clinical Trial Registry (ChiCTR1900021516). The patients recruited here were American Society of Anesthesiology (ASA) physical status I or II, aged from 18 years or older. They all had no known allergy to the drugs in this study. Exclusions included patients who might have chronic use of analgesic or sedative drugs or opioids, psychological problems, smoking history, facial pain, known psychiatric illness, diabetes, severe liver or renal function (ALT exceeding 3 times the upper limit of normal as an indicator of liver damage and serum creatinine and urea nitrogen exceeding the upper limit of normal values and are considered indicators of impaired renal function), asthma, sleep apnea syndrome, ischemic heart disease, a clinical history, or electrocardiographic evidence of heart block. The patients who were pregnant or refused to participate were excluded as well.

### 2.2. Method of Sedation

With the use of a computer-generated random list sealed and envelope technique, sixty patients arranged to be administrated dexmedetomidine or midazolam were split into two groups in a random manner. In each group, thirty patients were recruited. Researchers who were not directly participating in patient care made preparation for the infusions, while anesthesiologists, dentists, and patients were unaware of the distribution of drugs and between groups. Patients were monitored by electrocardiogram, pulse oximeter, and noninvasive blood pressure (BP) readings and were given 2 L/min of oxygen via oxygen insufflations. Patients in group M were given midazolam (0.05 mg/kg), followed by a continuous infusion of a midazolam (0.04-0.2 mg/kg/h) until the end of the surgery. Patients in group D received 1.0 *μ*g/kg of dexmedetomidine over a 10 min period, and then dexmedetomidine was continuously perfused (0.2-0.7 *μ*g/kg/h) until the end of the surgery. Ten minutes after the start of the loading dose, patients underwent local anesthesia with 1/100000 adrenaline and 4% articaine hydrochloride, under the administration of dentists. When anesthesia begins, appropriate surgery is performed by the same surgeon for all patients. If respiratory depression occurs, a simple breathing apparatus connected to oxygen was used to immediately assist breathing manually, and the sedative and analgesic doses were adjusted until respiratory depression disappears. We regarded systolic pressure < 90 mmHg (1 mmHg = 0.133 kPa), diastolic pressure < 60 mmHg, heart rate < 50 beat/min, or the changes in blood pressure and heart rate greater than 30% compared with baseline as adverse events of hypotension and bradycardia. Once the above situation occurs, relevant vasoactive drugs were used to maintain haemodynamic stability, and these cases would be excluded from the trial.

### 2.3. Observation Indicators

One of the investigators was not directly involved in the care of the patients and rated the study variables. A record of all indices was made before initiating sedation (i.e., baseline) and subsequently at the intervals of 15 minutes until 3 h after the drug was infused. The duration of surgery, ASA grade, sex, body weight, age, and dosage of local anesthesia in both groups were recorded. We measured heart rate (HR), arterial oxygen saturation (SpO_2_), diastolic blood pressure, and systolic blood pressure. The OAAS scores were assessed for sedation levels (Table 1), and the visual analogue scale (VAS) pain scores evaluated the level of pain on scales of 0–10 (0 being no pain and 10 being the worst pain). Likewise, surgeon and patient satisfaction levels were also rated on scales of 0–10 (10 means very good and 0 means very poor). At the end of the operation, patients were admitted into the anesthesia recovery room. A modified Aldrete score of 10 was the hospital discharge criterion. We asked patients to rate their satisfied levels of the extent of sedation and analgesia they received and the time consumed for eliminating the analgesia after their arrival at home. One analgesic tablet containing 500 mg of paracetamol was prescribed to the patients. If the postoperative visual analogue scale (VAS) pain score was more than 3, the patients were advised to take the pain medication prescribed.

### 2.4. Statistical Analyses

Before the study, with stable haemodynamics as the primary outcome, we determined that after 120 min of continuous infusion, a SBP of 130 ± 19 mmHg dropping to 104 mmHg was of clinical importance (*a* = 0.05, power = 0.8). The analysis showed that 16 subjects per group would be sufficient to detect a difference between the two groups. Assuming a 10% drop-out rate, the final sample size was set at a minimum of 18 patients per group. Continuous variables with a normal distribution are expressed as mean and standard deviation (SD). By Student's *t*-test, differences in SpO_2_, HR, and SBP values, age, weight, duration of surgery time, patient satisfaction score, surgeon satisfaction score, OAAS score, and VAS pain score were analyzed. Gender was analyzed using the *χ*^2^ test. A *p* value below 0.05 was considered of statistical significance for all tests.

## 3. Results

### 3.1. Patients

**Figure 1:**
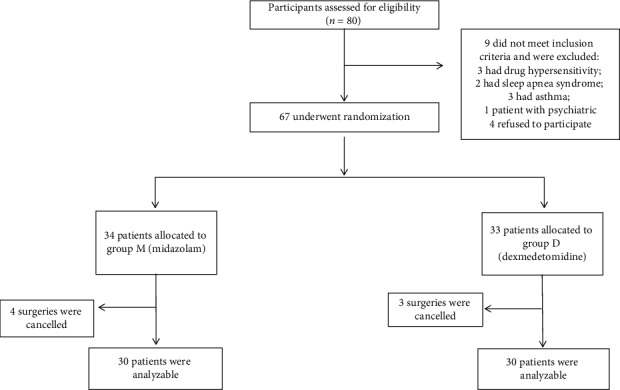
The flow diagram of the study.

Of the 80 participants assessed for eligibility, 9 of these patients did not meet the inclusion criteria and were excluded and 4 patients refused to participate. Then, 67 patients were randomly assigned to two groups (M, *n* = 34, and D, *n* = 33) by use of a computer-generated randomization list. However, there were 4 in midazolam and 3 patients in the dexmedetomidine group who were ruled out due to the surgeries being cancelled. 60 patients completed the analysis, with 30 subjects in each treatment group, as illustrated in the flow diagram (Figure 1).

Between the two groups, the total volume of local anesthetic used, duration of operation, surgical characteristics, and demographic data were not significantly different. The total time of analgesic effect was longer in group M than in group D (5.18 h compared with 3.92 h, *p* ≤ 0.001; Table 2).

### 3.2. SpO_2_, RR, and Haemodynamic Effects

**Figure 2:**
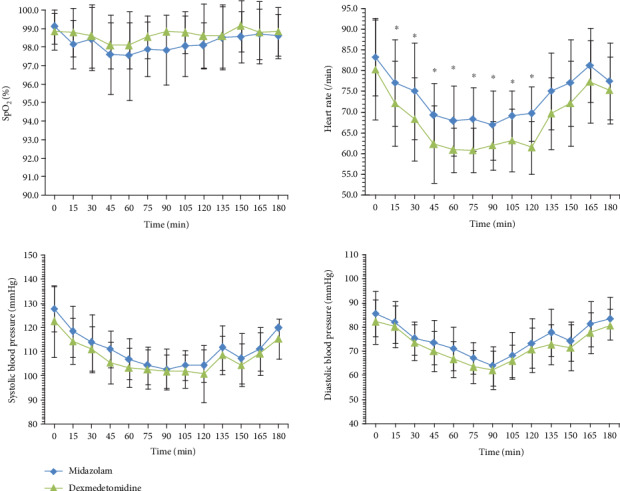
Saturation of pulse oxygen, heart rate, and systolic blood pressure values (mean ± SD) for the two treatment groups (group M and group D) during the course of 180 min. Time 0 min is before drug administration.^∗^*p* < 0.05.

Although the mean SpO_2_ of group M was lower, there was no difference between groups D and M. HR was lower in group D than in group M at 15–120 min after the administration of drugs; this difference was significant, except at 0, 135, 150, 165, and 180 min. The SBP of groups D and M was not significant. (Shown in Figure 2).

### 3.3. Analgesic and Sedative Effects

**Figure 3:**
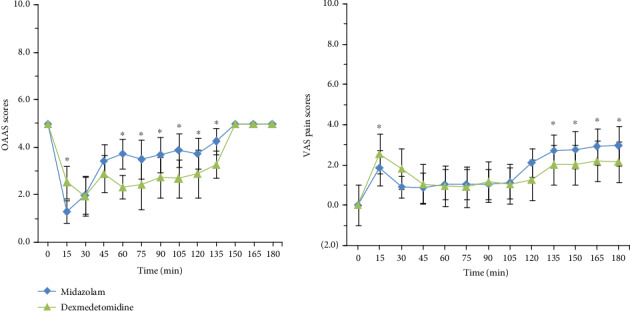
OAAS and VAS pain scores (mean ± SD) for the two treatment groups (group M and group D) during the course of 180 min. Time 0 min is before drug administration. ^∗^*p* < 0.05.

OAAS scores and VAS pain scores in both groups recorded at 15 min intervals until 3 h after drug infusion was conducted are shown in Figure 3. Patients administered dexmedetomidine had obviously lower OAAS scores than those receiving midazolam at 30–120 min after administration. Specifically, the OAAS score was higher in group D than in group M at 15 min. At 30–120 min, there was no statistical difference in the VAS pain scores in group D and group M. The VAS pain scores were significantly higher at 15 min and lower at 135 min, 150 min, 165 min, and 180 min, after the start of infusion in group D when compared with group M (Figure 3). More patients in group M had delirium after surgery. The incidences of other common side effects such as tiredness, headache, and nausea are similar between the two groups. No serious adverse events occurred in this study, and all patients had no postoperative complications.

## 4. Discussion

In this study, we compared the efficacy and safety of DEX and midazolam as sedatives in outpatient dental implant surgery under local anesthesia. The results showed that the DEX group was superior to the midazolam group in terms of sedation and postoperative pain relief.

Although some research reports significant haemodynamic variations at initial loading, it has been verified that a dose-dependent reduction in blood pressure and heart rate results from using dexmedetomidine [10, 11]. In this study, by infusing the drug over a 10 min timeframe slowly, such reduction was made minimum. There was no significant difference in SBP between the two groups, while the mean heart rate of the dexmedetomidine group was significantly lower. All the patients did not need interventional treatment, and the dental operation was successfully completed with no complication occurring. The intervention criteria were either respiratory (apnoea or desaturation or both) or cardiovascular (obvious bradycardia or hypotension).

Through titration, dexmedetomidine can reach the required sedation level without significantly inhibiting respiration. Midazolam often leads to the inhibition of respiration, whereas in this study, there was no significant difference in SpO_2_ between the two groups, and the drug did not significantly inhibit respiration. According to Hall et al. and Ustun et al., in the course of the operation and the recovery, sedation with dexmedetomidine preserved the respiratory rate and oxygen concentration [12]. The results of this study are consistent with those of these researchers, although respiratory inhabitation related to dexmedetomidine was not obviously lower than that related to propofol [13]. According to their consideration, the variations in tidal volume and arterial blood carbon dioxide tension require investigation.

Group D had obviously lower scores of OAAS sedation at 30–120 min. In group M, some patients were suffering pain after the operation time exceeded 30 minutes, which might be caused by the weakened anesthetic effect. This suggests lower sedation levels than in group D. This is largely because midazolam takes 30 to 60 sec to take effect and plasma concentration takes 5 min to peak. Nevertheless, the onset time of dexmedetomidine takes 10∼15 minutes to take effect, and plasma drug concentration takes 25∼30 min to peak. There was no statistically significant difference in the VAS pain score between group D and group M after 30-120 minutes of administration, whereas the VAS pain score was less than that of group M after 120-240 minutes of administration. As the local anesthetic was gradually eliminated, the pain became more significant. Dexmedetomidine has an analgesic effect, whereas midazolam does not. Midazolam takes a shorter time than dexmedetomidine when taking effect. For this reason, the VAS pain score was lower in the M group than in the D group after 15 min of administration. According to Dere et al. [14], compared with 0.05 mg/kg midazolam, a loading dose of 1 mg/kg dexmedetomidine and a maintenance dose of 0.5 mg/kg/h had better sedative effect. Dexmedetomidine can help achieve sedation, memory retention, and analgesia effectively without causing any cardiorespiratory complications, even at low doses (0.2 or 0.6 mg/kg) [15].

## 5. Conclusions

To sum up, compared with midazolam, dexmedetomidine has a better sedative effect in outpatient dental implantation under local anesthesia. Dexmedetomidine was found to be more effective in surgeon satisfaction, postoperative analgesia, patient-surgeon collaboration, and intraoperative arousal during office-based unilateral impacted tooth extraction. This study supports the safe use of dexmedetomidine in this group of patients, but further studies would need to be undertaken before the technique can be applied to the wider population.

## Figures and Tables

**Table 1:** Observer's assessment of alertness and sedation using the Observer's Assessment of Alertness/Sedation Scale.

Responsiveness	Speech	Facial expression	Eyes	Score
Responds readily to normal tone of voice	Normal	Normal	Clear, no ptosis	5
Responds slowly to normal tone of voice	Mild slurring	Mild relaxation	Mild ptosis, less than half the eye	4
Responds only after loud or repeated calling	Slurring	Pronounced relaxation	Glazed, obvious ptosis	3
Responds after mild prodding or shaking	Few recognised words	Pronounced relaxation	Glazed, obvious ptosis	2
No response to mild prodding or shaking	No words	Pronounced relaxation	Glazed, obvious ptosis	1

**Table 2:** Comparison of demographic information, clinical characteristics, and postoperative data of patients for the two groups.

Variables	Group D (*n* = 30)	Group M (*n* = 30)	*p* value
Age (year)	41.61 ± 9.82	43.34 ± 8.43	0.316
Body weight (kg)	61.12 ± 8.63	59.20 ± 7.73	0.168
Males/females	19/11	18/12	0.070
Duration of surgery (min)	117.40 ± 15.18	115.75 ± 13.57	0.719
Number of dental implants	2.35 ± 0.88	2.00 ± 0.73	0.177
Total volume of local anesthetic used (mL)	7.33 ± 0.67	7.48 ± 0.99	0.579
Surgeon satisfaction score	7.45 ± 1.15	7.60 ± 1.05	0.668
Patient satisfaction score	9.40 ± 0.59	9.25 ± 0.55	0.414
Time elapsed before taking the paracetamol tablet (h)	3.92 ± 0.49	5.18 ± 0.65^∗^	≤0.001

M: midazolam; D: dexmedetomidine. Data shown are the number or mean ± standard deviation. ^∗^*p* < 0.05.

## Data Availability

The data used to support the findings of this study are available from the corresponding author upon request.
